# Cell-Free Approaches in Synthetic Biology Utilizing Microfluidics

**DOI:** 10.3390/genes9030144

**Published:** 2018-03-06

**Authors:** Samar Damiati, Rami Mhanna, Rimantas Kodzius, Eva-Kathrin Ehmoser

**Affiliations:** 1 Department of Biochemistry, Faculty of Science, King Abdulaziz University (KAU), Jeddah 21589, Saudi Arabia; 2 Biomedical Engineering Program, The American University of Beirut (AUB), Beirut 1107-2020, Lebanon; rm136@aub.edu.lb; 3 Mathematics and Natural Sciences Department, The American University of Iraq, Sulaimani, Sulaymaniyah 46001, Iraq; kodzius@envirola.com; 4 Faculty of Medicine, Ludwig Maximilian University of Munich (LMU), 80539 Munich, Germany; 5 Faculty of Medicine, Technical University of Munich (TUM), 81675 Munich, Germany; 6 Department of Nanobiotechnology, Institute for Synthetic Bioarchitecture, University of Natural Resources and Life Sciences, 1190 Vienna, Austria; eva.sinner@boku.ac.at

**Keywords:** synthetic biology, microfluidics, de novo gene synthesis, cell-free protein synthesis

## Abstract

Synthetic biology is a rapidly growing multidisciplinary branch of science which aims to mimic complex biological systems by creating similar forms. Constructing an artificial system requires optimization at the gene and protein levels to allow the formation of entire biological pathways. Advances in cell-free synthetic biology have helped in discovering new genes, proteins, and pathways bypassing the complexity of the complex pathway interactions in living cells. Furthermore, this method is cost- and time-effective with access to the cellular protein factory without the membrane boundaries. The freedom of design, full automation, and mimicking of in vivo systems reveal advantages of synthetic biology that can improve the molecular understanding of processes, relevant for life science applications. In parallel, in vitro approaches have enhanced our understanding of the living system. This review highlights the recent evolution of cell-free gene design, proteins, and cells integrated with microfluidic platforms as a promising technology, which has allowed for the transformation of the concept of bioprocesses. Although several challenges remain, the manipulation of biological synthetic machinery in microfluidic devices as suitable ‘homes’ for in vitro protein synthesis has been proposed as a pioneering approach for the development of new platforms, relevant in biomedical and diagnostic contexts towards even the sensing and monitoring of environmental issues.

## 1. Introduction

Synthetic biology is a rapidly growing field that covers several disciplines including molecular biology, chemistry, physics, mathematics, engineering, and nanotechnology. The power of synthetic biology derives from the inspiration and diversity of living biological systems. The unique property of synthetic biology is the ability to design and build new semisynthetic systems to modify the system’s functionality or to perform new functions that the biological system is not able to perform naturally to meet desired needs [1,2,3]. Rapid advances in DNA sequencing and gene technologies have a direct impact on the progress of synthetic biology in terms of the ability to reprogram the cell [4]. However, the molecular toolkit for synthetic biology includes RNA, DNA, and proteins. Two to ten molecules from this toolkit are typically assembled in order to build a system that is able to perform the desired function when transplanted into a seminatural context. This construction process is complex as it needs genetic reprogramming, DNA and protein synthesis, and functional screening [5]. Understanding how life works is the main challenge in synthetic biology. The complexity of the biological systems and lack of knowledge make it difficult to mimic living systems. Indeed, uncontrolled interference between native and artificial components may affect the behavior of the artificial system by making it different to the biological one.

Synthetic biology research involves basically four points: (1) designing an artificial system, which usually is inspired from nature; (2) system construction using biological and nonbiological elements; (3) testing the developed system to confirm its functionality; and (4) analysis to determine the degree to which the manipulated system mimics the functional and structural properties of the natural system [5]. Recent research in synthetic biology has focused on the construction of genetic circuits, biological modules, and synthetic pathways in genetically reprogrammed organisms [1,3]. Combining two or more fields provides promising opportunities for diagnosis, development of genetic processing devices, high-throughput analysis of mutant libraries, high-resolution single cell analysis, and isolation and characterization of mutant or rare microbes or genes [6,7,8].

Integration of multidisciplinary techniques, such as de novo DNA synthesis, cell-free protein expression, and microfluidics, could contribute significantly to the field of synthetic biology by saving time and cost as well as improving the functionality of a developed system by making it robust and effective. In this review, cell-free gene and protein synthesis systems and their applications in microfluidic technology are discussed. The basic principles of cell-free protein expression systems are described with emphasis on the most commonly used systems. Constructing artificial cells from the bottom-up approach using biological and nonbiological building blocks, such as lipids and proteins, as well as using cellular machinery integrated with a microfluidic platform are also reported. This work presents microfluidic technology and its applications in synthetic biology as a promising platform to simplify cellular structure and functions, which can be used for engineering biological parts at protein and cell levels.

## 2. Cell-Free Protein Synthesis

**Figure 1:**
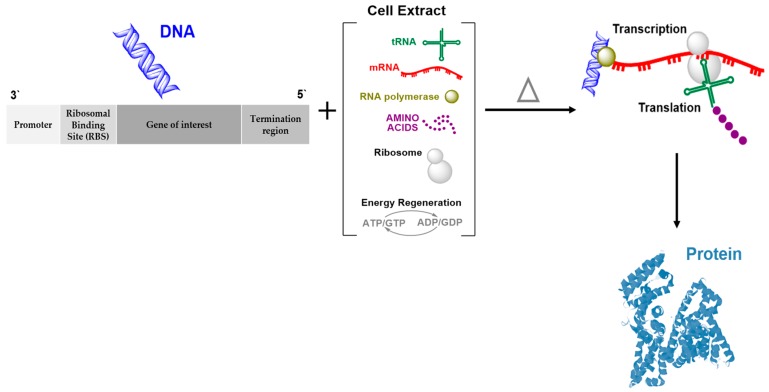
Cell-free protein expression system. The system requires a genetic template, e.g., DNA, that is composed of a promoter, a ribosomal binding site (RBS) which is either a Shine–Dalgarno or Kozak sequence, and a translation–transcription termination region. The reaction needs cell lysate and an energy regeneration system.

Cell-free (also known as in vitro) protein synthesis technology is a powerful alternative system to the cell-based (in vivo) system and exploits the cellular protein machinery to synthesize proteins directly outside living cells [9,10,11]. Table 1 summarizes the advantages and disadvantages of both systems. In the cell-free system, mixing exogenous mRNA or DNA with crude extract or lysate from any cell origin provides easily controllable transcription–translation machinery in an open environment (Figure 1). Several organisms have been used to prepare a lysate that contains all necessary enzymes and cellular factors needed for the protein expression machinery. Exogenous supplies of nucleotide, salts, essential amino acids, and energy-generating factors are also needed to develop a cell-free system [9,12]. The common commercial lysates available are *Escherichia coli*, yeast, rabbit reticulocyte, wheat germ, and insects (Table 2). The choice of extract source depends mainly on the origin of the desired protein to be expressed, its complexity, and further downstream applications. This choice is important and should be carefully considered as it affects the quantity of protein and generation of incomplete polypeptides or full-length proteins [12,13]. Another important factor that can significantly affect protein expression is the use of genetic templates. In coupled systems, DNA is used as a template encoding the target protein, while the mRNA template generated by in vitro transcription or from native sources is used in uncoupled systems. A plasmid or polymerase chain reaction (PCR) fragment can be used as a DNA template, although this must contain a promoter, such as T3, T7, or SP6, and a ribosomal binding site (RBS) that acts as a translation initiation signal, such as a Shine–Dalgarno or Kozak sequence, to generate prokaryotic or eukaryotic proteins, respectively [9].

The cell-free protein synthesis system offers many advantages compared with the cell-based systems, including high protein yield; generation of soluble and functional proteins without inhibition of regulatory pathways; the possibility of using mRNA or PCR fragments directly without any need for cloning; and the possibility of expressing multiple templates, which permits the production of the protein population in a single reaction [9,12,13]. Many proteins are unstable, proteolytically sensitive, or toxic, while in other cases, living cells cannot tolerate these proteins physiologically. Such proteins cannot be synthesized by a cell-based system, although they can be synthesized with the cell-free system [19,20]. Moreover, in the cell-based system, many proteins need post-translational modification to generate active proteins. In contrast, the cell-free system allows the addition of helper molecules which will improve protein folding and functionality. For example, dithiothreitol (DTT) is usually added to the cell-free system to increase protein expression and to preserve the cytoplasmic environment. DTT slightly affects the folding of cytoplasmic proteins and significantly affects the folding of proteins that form disulfide bonds for activity. Furthermore, the open nature of this system allows the incorporation of non-natural or chemically modified amino acids at specific positions during translation, which leads to the generation of novel proteins [9,17].

Apart from the crude extract containing the basic translation–transcription components, the protein synthesis using recombinant elements (PURE) system consisting of reconstituted, purified elements derived from the *E. coli* translation machinery is another option to express cell-free proteins. The PURE system reconstitutes the translation–transcription process from 31 purified recombinant proteins, 46 tRNAs essential substrates, and corresponding enzymes [18,19]. This system is adapted for in vitro protein selection and for library display. However, time, cost, and incomplete understanding of fundamental biology restrict the construction of the PURE system. Matsuura et al. [25] designed a computational model to investigate the dynamic features of large-scale biological reaction networks based on *E. coli*-based reconstituted in vitro protein synthesis. The developed model synthesized a peptide (Met-Gly-Gly) based on the components of the PURE system and kinetic parameters collected from the literature to simulate the reaction. The obtained model consisted of 241 components and 968 reactions and achieved a steady state in <1 min. Reyes et al. [26] designed a set of T7 promoters to express the protein in reconstituted and *E. coli*-extracted-based cell-free systems at different transcriptional rates. The expression level and the rate of protein production in response to transcription rate change were different in the two cell-free systems. Indeed, a simple mathematical model for the two cell-free systems was constructed to confirm that the changes in the transcription rate are driven by different expression dynamics. Several limiting factors that affect protein expression and reproducibility of the experimental data were investigated in this model. For the cell-free protein synthesis system, unwanted activities during protein synthesis are the main disadvantage of this system [1,27]. Continuous protein production is limited by the supply of exogenous materials, such as amino acids and nucleotides, and the accumulation of products. 

There are different cell-free protein synthesis reaction formats; the main two modes are batch and dialysis. The batch reactions are based on performing cell-free transcription/translation reactions in one vessel containing all the required elements. Although this mode is easy to handle, fast, and scalable, it has short reaction times which lead to limited protein production (micrograms per mL) due to component degradation and inhibitor accumulation, such as free phosphate and Mg^2+^ [28]. In contrast, the cell-free continuous-exchange mode can be optimized to prolong the reaction lifetime and increase protein production (milligrams per mL). In this reaction, transcription/translation coupled reactions are performed in one chamber but are separated from the reaction reservoir by a semipermeable dialysis membrane with a cutoff at 10–15 kDa. The reservoir continuously feeds the chamber with low-molecular-weight substrates including nucleotides, amino acids, ions, and energy resources and allow dilution of the by-products from the reaction chamber via the dialysis membrane, extending the reaction lifetime [20,29,30,31]. Different reactors in the integrated dialysis system of cell-free protein synthesis have been used including a dialysis bag clamped at both ends [32] and commercial devices from several suppliers, such as a rapid translation system (RTS) cell-free kit from Roche [33] and DispoDialyzer from Spectrum Laboratories, Inc. [34]. 

In synthetic biology, the cell-free protein expression system is exploited to construct membrane proteins in view of artificial cellular components, such as protein-functionalized membranes. Damiati et al. [35] developed one such approach in this direction, aiming to reconstitute the voltage-dependent anion channel (VDAC) protein into a synthetic membrane. The VDAC proteins were synthesized by a bacterial cell lysate, either in direct contact with a planar synthetic bioarchitecture of a membrane or synthesized and purified before exposure to the lipid membrane. The favorable surface-to-volume ratio in a microfluidic system had been previously investigated by our group [36,37,38,39]. The formation of the artificial membrane and reconstitution of the VDAC protein was monitored by quartz crystal microbalance with dissipation monitoring (QCM-D) and electrochemical impedance spectroscopy (EIS). In the first strategy, VDAC genetic materials were mixed with the translation–transcription machinery reaction mixture in the presence of the artificial membrane in order to allow immediate incorporation of the synthesized protein molecules once generated. QCM-D and EIS measurements showed degradation of the formed membrane architecture, which may be attributed to the interaction between the cell lysate and the synthetic membrane due to the molecular crowding of the cell-free extract mixture and the detergent-like environment. In contrast, purified VDAC proteins expressed by the cell-free system can be incorporated into the lipid membrane, which forms active channels to allow ions to move across the membrane.

The limited lifetime of the translation activity is a disadvantage of cell-free protein expression system, which leads to low yields of protein expression. Hence, a continuous flow of the cell-free lysate is proposed as a solution. Thus, microfluidic technology offers a promising approach, since the microfluidic chamber can not only provide continuous provision of tRNAs but also an energy source, while removing the educts of synthesis and producing a planar membrane with de novo synthesized and folded membrane proteins in optimal (physical and chemical) conditions.

## 3. Microfluidic Technology

**Figure 2:**
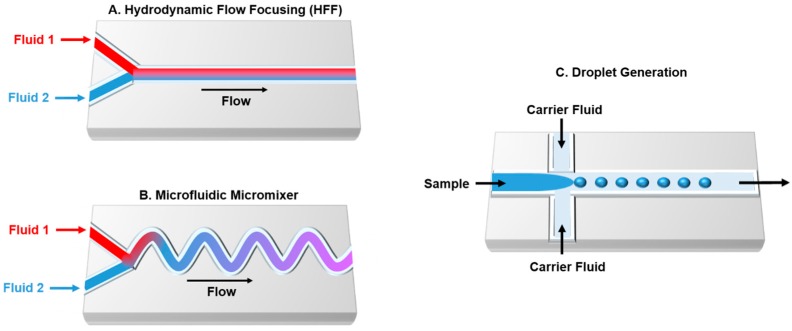
Examples of commonly used microfluidic techniques. (**A**) Microfluidic platform with hydrodynamic flow focusing; (**B**) microfluidic mixing chamber; (**C**) droplet-based microfluidic.

Microfluidic devices are widely used as a promising technology that allows analysis of molecular biology reactions and cell growth on the same platform. Compared with traditional, large-scale methods, microfluidics use fewer resources (minimum quantity of samples/reagents from microliters to femtoliters and less consumed), perform experiments rapidly and accurately, decrease cost, and enable high throughput and automation [40]. Careful design of microfluidic chips can effectively improve the throughput of the biochemical reaction and analysis. Controlling the geometry enables the subdivision of channels into multiple functional units, which involves mixers, reactors, detector, valves, and pumps. Furthermore, shorter microfluidic channels improve rapid heat and mass transportation [41]. Complex channel geometries promote mixing in microreactors, which leads to twisting, splitting, and recombining of fluid streams. Laminar flow is the major factor that controls the diffusion-controlled mixing of components at the interface of converging fluid streams. This is characterized by a low Reynolds (Re) number and organizes the flow rate of multiple fluids in parallel without turbulence in a single channel [42,43]. Several techniques are available that enable mixing of fluids and samples in microfluidic devices. Hydrodynamic flow focusing (HFF) is one of the most ultra-fast mixing methods that can be precisely achieve with a laminar mixer (Figure 2A) [44]. The main disadvantage of the diffusion-based microfluidics is related to the number of unreacted materials; this can be overcome by a microfluidic mixer (Figure 2B). Indeed, droplet-based microfluidics offers an alternative technique which allows independent reactions to occur within the droplets (Figure 2C) [45,46,47]. In synthetic biology, cells can be encapsulated into the generated droplets or the droplet itself can be exploited as a cell architecture. Thus, microfluidic devices are widely used to perform biological and chemical experiments [48,49]. Several materials are available to fabricate microfluidics devices, such as polymers (e.g., polydimethylsiloxane (PDMS)), metals, glass silicon, and ceramics. The material choice depends on the required temperature and pressure, utilization of corrosive fluids, and the desired application [50,51]. Furthermore, microfluidic chips can be designed by the integration of PCR machines, cell sorters, and detectors [52,53,54].

The availability of many microfluidic formats supports a variety of synthetic biology applications from DNA assembly to artificial cell construction [55]. Combining microfluidics with other fields, such as genetic, protein, and microbial engineering, adds value and offers many opportunities at advanced levels compared to the conventional approaches at point-of-care (POC) [6]. Indeed, the unique feature of the microfluidic scale is the simplification of the complex biological experiments by synthesizing de novo systems of interest.

## 4. Microfluidic Platforms and Cell-Free Applications

### 4.1. De Novo Gene Synthesis in Microfluidics

Microfluidic technology is proposed as a promising alternative to the traditional macroscale and microarray techniques for synthesizing oligonucleotides and genes with at least a tenfold improvement in performance [56]. Microfluidics is able to reduce the cost and time of DNA synthesis by integrating and parallelizing reaction chambers on a single device, minimizing reagents and labor in addition to enabling several rounds of buffer changes and rinses. An additional advantage of microfluidics is the ability to integrate each chamber with special capabilities (e.g., valves) to control the reaction easily [57,58,59,60,61]. Due to the incompatibility of polydimethylsiloxane (PDMA) with convenient DNA synthesis chemistry, microfluidic devices for de novo gene synthesis are usually formed or coated with resistant materials such as Teflon [62].

Microfluidics enables the accumulation of minimum quantities of genetic materials, which creates a single-cell resolution to study DNA sequencing and to analyze gene expression. An electrophoresis-based sequencing microchip was developed successfully by Fredlake et al. [63]. Compared with convenient capillary array electrophoresis instruments, the fabricated chip offered ultrafast DNA sequencing with an efficient approach for separating DNA fragments and reading lengths of up to 600 bases in 6.5 min. Lee et al. [58] reported on the successful fabrication of a microfluidic device that directly generates ~200 bp DNA constructs without any additional step for concentrating or amplifying the genetic materials. This device was able to reduce the reagent consumption by 100-fold and was able to synthesize ~100 pmol of the oligonucleotide sequence. Such direct synthesis reduces cost and time, while this method also eliminates the incorporation of errors into the full-length sequence during the amplification procedure. Another obstacle on the genome-scale solved by microfluidic devices is the limited quantity of the biological sample. Current microarray techniques for monitoring mRNA transcription levels need nanogram amounts of total RNA. Irimia et al. [64] fabricated a microfluidic device which enables RNA preparation and gene expression from a biological sample that contains ~150 cells. All steps, including cell lysis, RNase inactivation, and DNA and protein digestion, were performed on a single chip and the extracted material was enough for further transcriptome analysis. 

Traditional de novo gene synthesis techniques usually yield a pool of DNA fragments with limited sizes and require further assembly steps to ligate these short fragments into larger constructs [65,66]. On the contrary, microfluidics technology enables the rapid mixing of a selection of gene fragments and assembly of reagents in separated droplets to generate combinatorial libraries. A digital microfluidic device developed by Yehzekel et al. [67] was used for de novo synthesis, combinatorial assembly, and cell-free cloning of DNA libraries in submicroliter reaction droplets (300 nL) through subsequent additions. In this system, nine oligonucleotides were used as building blocks and approximately 160 bp of synthetic DNA was added to the construct during each polymerization step. Another system composed of eight DNA fragments and dispensing valves enabled the generation of 1 nL droplets that contain one type of the DNA fragments. Droplet pairs were collected and dispersed into separate wells of a microplate and were subsequently merged by centrifugation. These fragments were assembled by Gibson assembly. This technique was able to generate a 16-plex combinatorial library that was verified by PCR [68]. Ochs et al. [69] developed a single-layer pincher valving system. In this system, the microfluidic chip involves several valves that precisely regulate the fluid flow in channels to control the mixing of four different DNA fragments. Subsequently, this allows the formation of droplets. The encapsulation of DNA fragments into the droplets occurs with the addition of Golden Gate assembly reagents and droplets collected in a PCR tube, which undergoes further assembly reaction. Two microfluidic devices used modern DNA assembly methods (Gibson and Golden Gate). These methods have the ability for on-chip DNA construction because they omit any additional washing steps or subsequent addition of reagents [45]. The microfluidics of droplets has been exploited as an alternative to whole-genome sequencing, in which the genomic region of interest can be targeted and amplified for further sequencing. Tewhey et al. [70] developed a microfluidic droplet system which can perform up to 1.5 million PCR reactions in parallel, presenting a significant improvement in the experimental throughput.

The successful construction of DNA depends mainly on the quality of DNA fragments, required purification, and assembly processes. Hence, further efforts are needed to develop microfluidic devices that allow preparation, synthesis, purification, and assembly on a single chip. The fully integrated system could be a promising research tool for gene construction and screening. 

An alternate, versatile platform for synthetic biology, paper-based synthetic gene networks were developed by Pardee et al. [71] for in vitro applications in health, clinics, research, and industry. In this model, the enzymes of transcription/translation reactions were combined with engineered gene circuits. Subsequently, the cell-free synthetic gene networks were embedded onto cellulose paper by freeze-drying pellets of the cell-free expression system and other porous materials which can be activated later by a rehydration reaction. The fabricated circuit was a low-cost, electronic optical interface that can be detected colorimetrically by the naked eye. Indeed, the resulting abiotic paper-based platform is a stable, sterile, and portable synthetic gene network external to the cell. Paper-based microfluidics can be even made more complex, utilizing wax- [72] or glue-assisted bonding [73]. The possible enzyme and reaction component interactions with the microfluidics components and the biocompatibility of the material should be taken into account [51].

### 4.2. Microfluidic Cell-Free Protein Synthesis

The combination of microfluidic technology with cell-free protein synthesis systems improves the protein synthesis and characterization in parallel to synthetic biology. However, the rapid growth of genome sequencing has attracted more attention, with a focus on finding the relationship between a gene and its corresponding protein. Hence, microfluidic devices have been proposed as a promising environment for the post-genome era, allowing us to run more experiments compared with utilizing the genome alone [74,75]. The investigation of a protein synthesized by a newly discovered gene is based on the availability of a sufficient quantity of the protein. Microfluidic channels and the open nature of cell-free systems address this problem by providing a continuous supply of nutrients (e.g., nucleotides, amino acids) and removing by-products. As published by Khnouf et al. [76], a continuous exchange cell-free protein synthesis device consisting of 96 units to express β-glucuronidase was fabricated. The solution continuously fed into the microfluidic device increased protein production by up to 87 times compared with the traditional microplate. Apart from reducing the consumption of reagents, microfluidic devices allow drug screening and further analysis on the same chip without any requirement for protein harvesting [76]. A cell-free system with an affinity assay integrated with a microfluidic platform was developed for large-scale investigations of protein–protein interactions by Gerber et al. [77]. In this system, the interactions of 43 *Streptococcus pneumoniae* proteins were investigated, with this experiment performed in quadruplicate. A total of 14,792 experiments were performed on the same chip, resulting in the discovery of new physical interactions in the biochemical pathways.

The integration of a cell-free protein synthesis system with microfluidic chips can be exploited for therapeutic (produce therapeutic agents) or diagnostic (detect toxins, antigens, cells) purposes. The generation of therapeutics at the POC, which will provide costly drugs in their pure form, such as orphan drugs, and personalized medicine, is currently needed [78]. Timm et al. [79] designed a microfluidic bioreactor, which allows the generation of a single dose of a therapeutic protein. This footprint device integrates a nanofabricated membrane which enables the exchange of materials between the cell-free protein reactor and feeder channels to enhance the exchange of energy, metabolites, and inhibitors. This device improved protein expression quantity and protein rate expression. For toxin detection, Hugh et al. [80] developed an array device to detect toxins. This device was composed of three units, with each one consisting of a reaction chamber and a feeding chamber for in vitro synthesis of three proteins (green fluorescent protein, chloramphenicol acetyl-transferase, and luciferase). High levels of protein expression were noticed due to the continuous feeding, removal of small by-products, and absence of inhibitors. When toxin simulants (either tetracycline or cycloheximide) were added, protein production yield was decreased. Indeed, the fabricated device was used to detect ricin, a bioterrorism agent that inhibits human protein expression. Ricin has been identified as inhibiting the production of luciferase. Synthesis of luciferase into the microfluidic chamber was indicated by luminescence. Luciferase production was decreased with increasing ricin concentration in the microfluidic channel.

Since the cell-free protein synthesis system is not restricted by physical barriers, it can be merged with sensor devices for real-time analysis and sensitive characterization of protein functions. Monitoring techniques are based on fluorescent labeling, optical microcavities, mechanical resonators, or nanowires [81]. Fluorescent resonance energy transfer (FRET) and fluorescent correlation spectroscopy (FCS) are examples of fluorescent detection methods. Ridgeway et al. [82] developed a microfluidic system that uses FRET to monitor the binding kinetics of rRNA and a ribosomal protein. They also used FCS for a two-photon detection mechanism in order to minimize the photobleaching of the sample. Moreover, the integration of a cell-free protein system and microfluidic platform with surface plasmon resonance (SPR) allows in situ investigation of the kinetics of protein interactions in real time [83,84]. Due to the ability of SPR to detect the mass adsorbed on the surface, Lee et al. performed cell-free protein synthesis reactions on an SPR chip. The generated protein molecules were bound to the chip, which generated SPR signals in real time [85]. In another study using the dual elements of on-chip protein expression and capture methodology, a protein microarray from a double-stranded DNA (dsDNA) microarray in a microfluidic platform was developed and SPR was used for bio-sensing measurements [86]. 

A problem associated with cell-free protein synthesis systems is degradation and hydrolysis of RNA molecules. A microfluidic Transcription–RNA Immobilization and Transfer–Translation (TRITT) was developed to overcome this obstacle. The TRITT chip enables cell-free protein synthesis with quasi-continuous mRNA transfer under controlled conditions for in vitro translation and transcription in separated compartments [87].

### 4.3. Microfluidics and Proteomics

The ability of the cell-free protein synthesis system to generate protein populations in a single reaction has been exploited to develop proteomic tools, which have been used in protein discovery in vitro and selection from large, diverse libraries. A proteome is an entire set of proteins that is derived from the genome directly and is subsequently used to regulate gene expression and cellular metabolism. The quantitative analysis of a proteome helps us to understand biology at the system level [88,89]. Thus, employing microfluidic devices in proteomic research is widely used for preconcentration, separation, and single-cell proteomics [89].

An obstacle facing protein research is the generation of a low protein quantity, which can be solved by a preconcentration step to increase protein concentration to a detectable range. Kelly et al. [90] developed a membrane-integrated microfluidic chip that concentrated protein. For example, R-phycoerythrin exceeded the original amount of the protein by 10,000-fold to improve microchip capillary electrophoresis. Additionally, compared with convenient capillary-based systems, this electrical field gradient focused on microchip-enhanced proteins, which increased the resolution by threefold and enriched the florescent-labeled peptide by a factor of >150-fold. A flat nanofluidic filter within a microchannel was fabricated as a microfluidic sample preconcentration chip. This system is based on electro-kinetic trapping, in which the nanofluidic filter acts as an ion-selective membrane that traps charged molecules for several hours with a concentration factor of 10^6^–10^8^ fold [91]. Another system composed of a thin-walled PDMS section between two microchannels enabled the preconcentration of negatively charged proteins under an electronic field. Protein molecules were assembled on the anode with an elevated concentration of up to 10^3^–10^6^ fold [92].

To evaluate protein dynamic interactions, it is important to quantify the number of proteins present in the system, which helps us to study biology at the protein level. Microcapillary electrophoresis (µCE) is widely used to separate proteins [93,94]. Duffy et al. [95] designed a rapid prototyping PDMS-based µCE system in less than 24 h. This microfluidic chip allowed the separation of DNA fragments, amino acids, and charged ladders of positively and negatively charged proteins in aqueous solutions with a resolution that is comparable to other systems using fused silica capillaries. Two-dimensional (2D) CE was developed to improve the separation resolution by coupling two separation modes orthogonally [96]. Li et al. [97] developed an emerged protein concentration/separation system. In this model, non-native isoelectric focusing was combined with SDS gel electrophoresis on a polycarbonate microfluidic device. The overall peak capacity of the 2D protein separations was ~1700, which was obtained in >10 min.

Single-cell proteomics enables the characterization of protein expression in individual cells, which offers valuable insight for generation within a heterogeneous cellular population. Microfluidic platforms have attracted attention for single-cell proteomics research due to their ability for automation and their compatible sizes with cells [88]. McClain et al. [98] fabricated a microfluidic device that integrates cell handling, cell lysis, electrophoresis separation, and fluorescent detection on a single chip. The loaded cells were hydrodynamically transferred from a reservoir to a region where they were concentrated and then rapidly lysed by the pulses from an electric field within 33 ms. This microfluidic system showed a total separation time of 2.2 s. Ros et al. [99] reported on an integrated microfluidic chip with cross-shaped microchannels for the docking of single cells. Each cell was manipulated into the docking area by an optical tweezer before the cells were lysed by electrical pulses. The released protein contents were subsequently separated by electrophoresis and detected by laser-induced fluorescence. 

### 4.4. Artificial Cells in Microfluidics

Synthetic biology involves constructing/mimicking the biological system by merging different fields in order to generate artificial biomimetic cells by reducing complexity, which helps in understanding the origin of life. To construct artificial cells, two approaches from two opposing directions can be followed: top-down and bottom-up [100,101,102]. The top-down method starts from living cells and descends to generate cells with the minimal genome needed for survival. The main disadvantages associated with this approach include the difficulty in scaling up, time cost, and expensiveness [4,103]. In contrast, the bottom-up approach starts with simple biological and chemical components and ends with a cell composed of the minimal requirements for life with the ability to perform a desired function [104]. 

**Figure 3:**
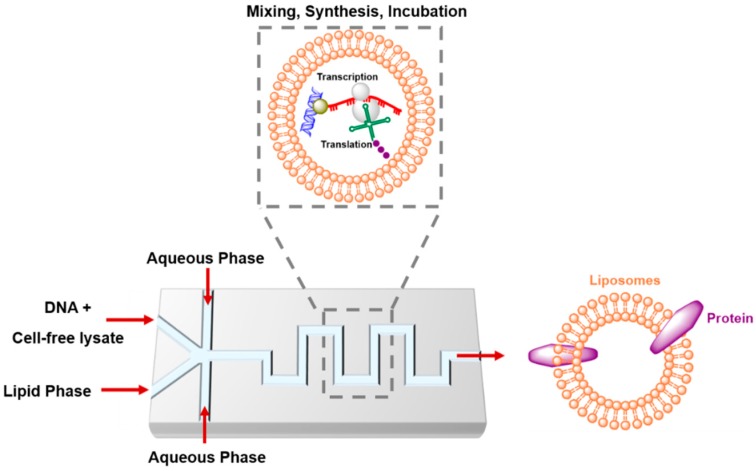
A strategy for the microfluidic generation of cell-free protein expression and direct reconstitution into lipid vesicle.

Integrating the bottom-up cell-free systems with microfluidic technology is becoming a powerful toolbox to engineer seminatural cells [105,106,107]. These cells are able to self-organize and self-reproduce to perform functions that exist in nature, such as protein synthesis, and can be used to investigate biological networks [108,109] or investigate novel functions, such as creating new signal pathways or synthesizing smart cells that deliver drugs on-site in response to stimulation [23,110]. The cell-free protein expression system, especially the PURE system, has been recently applied to artificial cell models. The integration of de novo DNA synthesis with replication in the artificial cells provides genetic information sustainability in the synthetic system [111]. A critical step in artificial cell construction involves compartmentalized translation–transcription machinery materials or any biological elements from the exterior environment. This must be conducted without a loss of function in order to preserve the genetic information and corresponding phenotype. The physical barriers can be based on water droplets, polymers, and lipid vesicles [112,113]. Hence, microfluidic technology allows bioinspired cell-like droplets to be generated and manipulated on chips (Figure 3). Droplet-based microfluidics offers several advantages, such as mono-dispersity, compartmentalization, high-throughput generation, and performing of functional operations in droplets [48,114,115]. The designed DNA template and cell-free protein expression system have been successfully encapsulated into droplets that are generated by microfluidic chips. For example, cell-free green fluorescent protein was expressed from a single molecule of DNA template into picoliter water-in-oil (W/O) droplets. The microfluidic reservoir chamber stored ~10^6^ droplets, which were stable for at least 6 h. This extends the time for further biochemical analysis [116]. A droplet-based microfluidic system was developed that enabled cell-free MreB, a structural membrane-associated protein, to maintain the rod-like shape of bacteria. This was encapsulated and expressed into single emulsions. The generated micrometer-scale protein patches were distributed at the water/oil interface [117]. Ho et al. [118] built a double-emulsion device to allow the combination of plasmid DNA and cell-free reaction mixture and subsequent synthesis of the mechanosensitive channel protein. This bacterial protein opens when the membrane is under high tension and decomposes into ultra-thin double emulsions. Since the cellular membrane has an asymmetric phospholipid bilayer that may allow lipid–protein interactions, Kamiya et al. [119] developed cell-sized asymmetric lipid vesicles to mimic biological membrane asymmetry by applying microfluidic flow to a micro-sized planar asymmetric lipid bilayer. Generation of giant vesicles with asymmetric lipid distribution and a low content of organic solvents plays a role in membrane protein reconstitution. Synthesis of in vitro membrane protein into giant asymmetric vesicles enhances the reconstitution ratio of the synthesized proteins. The differences in the reconstitution rate of membrane proteins into lipid bilayers may attributed to the electrostatic attraction between the protein and the asymmetric lipid membrane. The generated giant vesicles exhibited a lipid membrane flip-flop behavior similar to the lipid flip-flop activity noticed in apoptotic cells. 

Sokolova et al. [120] developed a platform based on W/O droplets that stimulates coacervation of *E. coli* cell lysate and following gene expression under crowded or noncrowded conditions. The phase separation of cell lysate into a lipid coacervate comprising complex mixtures of proteins and other macromolecules has allowed a direct comparison between mRNA synthesis in dilute and crowded environments. In a crowded environment, the binding constant of T7 RNA polymerase to DNA and the rate constant of transcription is significantly improved. The liquid–liquid phase transitions and production of crowded compartments significantly support in vitro transcription and translation. Hence, generation of an artificial cell-like environment by coacervation enables synthesis of mRNA at a high rate and reflects the crowding effects on key cellular reactions. This phase separation model needs to be exploited in microfluidic devices in order to mimic the regulatory mechanism of compartmentalization of biochemical reactions within the cells. Several features can be offered by the phase separation model for transcriptional control including dynamic changes, clustering of factors, production of super-enhancers, allowing simultaneous activation of multiple genes by the same enhancer, and enhancing the sensitivity of super-enhancers against transcriptional inhibitors [121]. 

Apart from the creation of droplets as a cell-like environment, microfluidic devices themselves can be used as artificial cells and are recognized as cells-on-a-chip. Karzbrun et al. [109] grafted DNA brushes on a microfluidic chip. After this, they allowed the continuous flow of the cell-free reaction mixture in the main channel, resulting in the continuous production of green fluorescent protein. Using the microfluidic chip as an alternative to vesicles enables the constant influx of building elements into the channels and collection of reaction products with a high degree of control. This system allows the study of biological networks in a simplified, controlled, and fully addressable seminatural environment. 

## 5. Conclusions

Synthetic biology can be defined as the reprogramming of an existing natural system to construct a seminatural one—even in the absence of living cells. The growth of synthetic biology as a field integrates high-throughput experiments and screening platforms. The current methods to engineer cells are costly, time-consuming, and difficult. Exploiting cell-free synthetic biology in combination with microfluidic technology will provide better engineering solutions and open more opportunities in different applications from basic (protein biochemistry) to biomedical science to biofuel research. Cell-free synthetic biology in genome, protein biochemistry, or proteomic fields is capable of investigating such biological materials by constructing a biosynthetic system without the membrane boundaries that account for low efficiencies and misfolding in the conventional cellular platforms. Indeed, the advanced design of the microfluidic platforms as an integrated system enables highly controlled gene and protein synthesis by robots that easily handle reagents/samples. Apart from the precise control, microfluidic devices allow real-time, rapid analysis at a small scale. Cell-on-a-chip offers a good model to provide modules, resembling the natural context, which may in future reduce the gap between in vivo and in vitro systems. 

## Figures and Tables

**Table 1:** Comparing between cell-free and cell-based protein expression systems [4,9,10,11,12,13,14,15,16].

Cell-Based System	Versus	Cell-Free System
Yes	Needs cloning	No
No	Ability to produce toxic protein	Yes
No	Ability to express multiple genes	Yes
No	Usually generate functional, soluble, and folded proteins	Yes
No	Possible adjusting and controlling by the addition of helper molecules	Yes
No	Possible incorporation of non-natural or chemically modified amino acids	Yes
Yes	Native environment	No
Days	Time	Hours
Low	Costs	High

**Table 2:** Comparison of different cell-free protein expression systems [9,12,17,18,19,20,21,22,23,24].

	*Escherichia coli* Extract	Rabbit Reticulocyte Lysate	Wheat Germ Extract	Yeast Cells, Tumor Cells, Insects
**Protein yields**	High (mg)	Low (µg)	High (mg)	Low (µg)
**Generated proteins**	Many incomplete polypeptides	Mainly full-length, folded proteins	Mainly full-length, folded, multidomain proteins	Mainly full-length, folded, multidomain proteins
**Translation modifications**	Post-translation	Co-translation	Co-translation	Co-translation
**Recommended template sources**	Bacteria	Prokaryotic (bacteria, mammalian virus, plant virus), Eukaryotic (plants, animals)	Prokaryotic (bacteria, plant virus), Eukaryotic (plants, animals)	-
**Genetic modification tools**	Well established	Poor	Poor	Poor
**Extract preparation**	Simple	Requires complex manipulation of animal tissues but cell breakage is easy and fast	Complex and time- consuming	Cell cultivation is complex and time-consuming, but cell breakage is easy and fast
**Cost**	Low	High	Low	High
